# Reduced biophotonic activities and spectral blueshift in Alzheimer’s disease and vascular dementia models with cognitive impairment

**DOI:** 10.3389/fnagi.2023.1208274

**Published:** 2023-09-01

**Authors:** Zhuo Wang, Zhipeng Xu, Yi Luo, Sisi Peng, Hao Song, Tian Li, Jiaxin Zheng, Na Liu, Shenjia Wu, Junxia Zhang, Lei Zhang, Yuan Hu, Yanping Liu, Dongwei Lu, Jiapei Dai, Junjian Zhang

**Affiliations:** ^1^Department of Neurology, Zhongnan Hospital of Wuhan University, Wuhan, China; ^2^College of Life Science, Wuhan Institute for Neuroscience and Neuroengineering, South-Central Minzu University, Wuhan, China; ^3^Medical Science Research Center, Zhongnan Hospital of Wuhan University, Wuhan, China; ^4^Department of Laboratory Medicine, Zhongnan Hospital of Wuhan University, Wuhan, China; ^5^Academy of Chinese Medical Sciences, Henan University of Traditional Chinese Medicine, Zhengzhou, China

**Keywords:** Alzheimer’s disease, vascular dementia, cognitive impairment, biophotonic activity, spectral blueshift, synaptosome, synaptic dysfunction

## Abstract

**Background:**

Although clinically, Alzheimer’s disease (AD) and vascular dementia (VaD) are the two major types of dementia, it is unclear whether the biophotonic activities associated with cognitive impairments in these diseases share common pathological features.

**Methods:**

We used the ultraweak biophoton imaging system (UBIS) and synaptosomes prepared by modified percoll method to directly evaluate the functional changes in synapses and neural circuits in AD and VaD model animals.

**Results:**

We found that biophotonic activities induced by glutamate were significantly reduced and spectral blueshifted in synaptosomes and brain slices. These changes could be partially reversed by pre-perfusion of the ifenprodil, a specific antagonist of the GluN2B subunit of N-methyl-D-aspartate receptors (NMDARs).

**Conclusion:**

Our findings suggest that AD and VaD pathology present similar but complex changes in biophotonic activities and transmission at synapses and neural circuits, implying that communications and information processing of biophotonic signals in the brain are crucial for advanced cognitive functions.

## 1. Introduction

Although AD and VaD have different pathological features and pathogenic mechanisms, synaptic dysfunction has been increasingly recognized as an early critical pathophysiological event in AD and VaD (Iadecola, 2013; Manczak et al., 2018; John and Reddy, 2021; Wu et al., 2021). As the primary excitatory neurotransmitter in central nervous system, glutamate has been proven by many studies to play an important role in AD and VaD pathology, and the glutamate antagonist memantine has been used in clinical practice to treat AD and VaD (Lipton, 2006; Reiner and Levitz, 2018). The excessive activation of glutamate receptors at synapses, especially GluN2B-containing NMDARs, is considered to be a key risk factor for synaptic dysfunction (Paoletti et al., 2013; Kodis et al., 2018). However, the detailed changes in synaptic function in these two diseases remain insufficient due to technical difficulties. Electrophysiological recording of long-term potentiation (LTP) and long-term depression (LTD) can be used to evaluate changes in postsynaptic potentials to a certain extent, but the low spatial and temporal resolution of membrane current and potential recordings, as well as tissue damage caused by electric shocks, pose challenges for directly assessing functional changes in synapses and neural circuits (Miller et al., 1996; Schuske et al., 2003; Guerrero et al., 2005; Fan et al., 2018).

Through the developed ultraweak biophoton imaging technology, our previous researches have found that the biophotonic activities induced by glutamate transmit along the neural circuits, suggesting that this new technology can be used to not only detect the biophotonic activities of neural circuits but also evaluate their functional changes (Sun et al., 2010; Tang and Dai, 2014a; Wang et al., 2016; Chai et al., 2018, 2022; Chen et al., 2020). We found that glutamate-induced biophotonic activities and transmission present a spectral redshift feature in the human brain compared with other animals (in order of bullfrog, mouse, chicken, pig, and monkey), while a spectral blueshift characteristic were observed in the brain of aging mice (Wang et al., 2016; Chen et al., 2020). It had also been demonstrated that unlike traditional techniques, the ultraweak biophoton imaging could be used to monitor delayed functional neural circuit injury in an animal model of cerebral ischemia-reperfusion (Chai et al., 2022). Moreover, our recent study combined the potential recording and biophoton imaging technology to discover the transsynaptic biophotonic activity and transmission caused by intracellular simulated biophoton stimulation (Liu et al., 2022). But, there is no study to directly assess changes in biophotonic activity and transmission at synapses.

In the present study, synaptosomes prepared from AD and VaD model animals were used to study functional changes in glutamatergic synapses and neural circuits through the biophoton imaging technology to provide new evidence for the pathogenesis of synaptic dysfunction in the development of AD and VaD.

## 2. Materials and methods

### 2.1. Animals

Six-month-old male triple-transgenic Alzheimer’s disease (3xTg-AD) mice (harboring the *PSEN1 M146V* knockin, *APPswe* and *TauP301L* transgenes) were purchased from the Jackson Laboratory (United States, RRID: IMSR_JAX:033930). Age-matched C57BL/6 J male mice were employed as wild type (WT) controls. Eight to 10-week-old Sprague–Dawley male rats were purchased from SPF Biotechnology Co., Ltd. (China).

The animals were allowed free access to water and food and housed in a room under standard conditions as follows: constant temperature of 22 ± 2°C, relative humidity of 55 ± 5%, and a light/dark cycle of 12:12 h. All animals used in this study were treated humanely according to the *Guide for the Care and Use of Laboratory Animals* published by the National Academy of Sciences and the National Institutes of Health. The animal protocols were approved by the Animal Ethics Committee of the Medical School of Wuhan University (No. ZN2021020).

### 2.2. Chronic cerebral hypoperfusion model

A CCH model was constructed by two-vessel occlusion (2VO). All rats were fasted for 12 h before the operation. After anesthesia with 1% Pelltobabitalum Natricum [40 mg/kg, intraperitoneal (i.p.) injection], the carotid arteries of the rats were carefully exposed. The rats in the 2VO group underwent double permanent ligation of the arteries with a 4-0 silk suture, while the rats in the sham group did not undergo ligation. The body temperature of the rats was maintained at 37.5 ± 0.5°C during the operation. Behavioral tests were carried out at 8 weeks after sham surgery or 2VO.

### 2.3. Cerebral blood flow

Blood flow in the left side of the hippocampal CA1 region (anteroposterior = 4.8 mm, mediolateral = ±2.5 mm, and dorsoventral = −3.5 mm) was measured by a laser Doppler probe (moorVMS-LDF1, UK) before and after sham surgery or 2VO (Supplementary Figure S1). Hippocampal blood flow is presented as the percentage of the postoperative value relative to the preoperative value.

### 2.4. Novel object recognition test

The NOR test was performed to evaluate the short-term memory. The experimental animals were allowed to explore the apparatus, i.e., an opaque open-field box (50 cm × 40 cm × 40 cm) in the absence of objects for 10 min for 2 days. In the first trial, two identical objects, namely, objects A and A′, were placed in the box, and the animals were allowed to explore for 10 min. One hour later, object A′ was replaced with object B, which was different from both A and A′. In the second trial, the animals were placed in the box again and allowed to explore for 5 min. The behavior of the animals was video recorded, and the time each animal spent exploring object A and object B was measured using a computerized video imaging analysis system [Animal Video Tracking Analysis System (AVTAS), China]. The discrimination ratio was calculated as previously reported: discrimination ratio (%) = T_B_ / (T_B_ + T_A_), where T_B_ represents the time spent in exploring the new object B and T_A_ represents the time spent in exploring the familiar object A.

### 2.5. Morris water maze test

The MWM test was performed to evaluate spatial memory. The MWM apparatus consisted of a cylindrical black pool (150 cm in diameter and 60 cm in height) filled with water and a black platform (10 cm in diameter and 30 cm in height) submerged 1 cm below the water surface. Visual cues were pasted above the walls of the pool, allowing the animals to learn the location of the platform. During the training period, i.e., day 1 to day 5, the animals were gently released into the water at four different locations on opposite sides of the platform. The animals were allowed to search freely in the water for 60 s until they found the hidden platform. Once an animal reached the platform, it was allowed to stay there for 15 s for acclimation to the surrounding environment. The time spent on the search platform was recorded as the escape latency, and the average escape latency over four trials was calculated. In the probe trial on day 6, the platform was removed, and the animals were allowed to swim in the water for 60 s. The time spent in the target quadrant was recorded. All data were acquired using a computerized video imaging analysis system (AVTAS, China).

### 2.6. Preparation of hippocampal synaptosomes

A rapid Percoll gradient method was used to prepare synaptosomes. In brief, fresh hippocampal tissues were homogenized in ice-cold sucrose/EDTA buffer (0.32 M sucrose, 1 mM EDTA, 0.25 mM DTT and 5 mM Tris, pH = 7.4). After centrifugation at 1,000 × g for 10 min, the supernatant (S1) was removed and then separated on a discontinuous Percoll gradient (3, 10, 15, and 23% vol/vol in sucrose/EDTA buffer). The samples were centrifuged at 31,000 × g for 5 min (Beckman Coulter Allegra 64R, United States), and fraction 4, which contained synaptosomes, was collected in sucrose as an isotonic solution. Care was taken to not disturb the gradient. Fraction 4 was diluted with ice-cold sucrose/EDTA buffer and centrifuged at 18,000 × g for 10 min at 4°C. The protein concentration was measured with a BCA kit (Thermo Fisher, United States), and the samples were diluted to a protein concentration of 5 mg/mL. The prepared synaptosomes were then gently resuspended in isotonic physiological buffer for subsequent experiments.

### 2.7. Preparation of brain slices

The brains were quickly removed from decapitated animals, and placed in ice-cold (0–4°C) ACSF. The ACSF consisted of 125 mM NaCl, 2.5 mM KCl, 2 mM CaCl_2_, 1 mM MgCl_2_, 1.25 mM NaH_2_PO_4_, 26 mM NaHCO_3_ and 20 mM D-glucose (pH = 7.4). Coronal brain slices contained the anterior part of the hippocampus (450 μm thickness) were sectioned consecutively by a Leica VT1000S vibratome (Leica, Germany). This step should be completed as soon as possible within 10 min to reduce brain tissue injury and degradation.

### 2.8. Biophoton imaging

The ultraweak biophoton imaging system BP-301 (Neukang, China) mainly consists of four parts, i.e., an EMCCD camera iXon Ultra 897 (Andor, UK) mounted with a Navitar’s high-speed fixed focal length lens DO-5095 (Navitar, United States), a stereomicroscopic supporter, a perfusion system and a dark box. The specific experimental methods were as follows:

1) Preparation of synaptosomes or brain slices: Coronal brain slices with a thickness of 450 μm or synaptosomes suspension diluted to a protein concentration of 5 mg/mL were prepared as described above.
2) Resuscitation of synaptosomes or brain slices: The prepared synaptosomes or brain slices were incubated in ACSF at room temperature for 1 h, and continuously aerated with a mixture of 95% O_2_ + 5% CO_2_.
3) Preparation of experimental drugs: Glutamate solution was dissolved in low osmotic pressure ACSF containing 50 mM glutamate, 115 mM NaCl, 2.5 mM KCl, 1.0 mM MgCl_2_, 26 mM NaHCO_3_, 1.25 mM NaH_2_PO_4_, 2.0 mM CaCl_2_ and 10 mM D-glucose (pH = 7.4), and ifenprodil (10 μM) was dissolved in ACSF.
4) Perfusion of synaptosomes or brain slices: Resuscitated synaptosomes or brain slices were transferred to a perfusion chamber in the dark box. The perfusion fluid was maintained through input and output peristaltic pumps (5 mL/min) outside the UBIS, and a mixture of 95% O_2_ + 5% CO_2_ was constantly supplied by a membrane oxygenator placed in the perfusion fluid. An electric heater was used to maintain the perfusion fluid in the perfusion chamber at a temperature of 37°C. The activity of synaptosomes and brain slices treated with perfusion can be maintained during subsequent biophoton imaging processes.
5) Image acquisition: The working temperature of the EMCCD camera was maintained at −90°C (water cooling mode), and then the image acquisition mode was adjusted to photon detection model 3, the EM gain was set to 1,200 × and the binning was set to 1 × 1. Real-time spatial images of biophotonic activity were obtained by automatically acquiring an image every 1 min for 540 min; images collected for the first 60 min were used to exclude the effects of ambient light, and images collected over the next 480 min were used for the analysis of biophotonic activity in the initiation, maintenance, washing, and reapplication periods.
6) Image processing and analysis: All original gray images in TIF format were first processed with a program on the MATLAB platform to eliminate the effect of cosmic rays (white spots) as previously described (Wang et al., 2016), yielding processed biophoton gray images. The average gray values (AGVs) in the regions of interest (ROIs) of the processed biophoton gray images and the background average gray values were extracted with an image analysis software program (Andor Solis for Imaging Version 4.27.30001.0; UK). The relative gray values (RGVs) of biophoton activity were calculated as follows: RGVs = AGVs (ROI area) - AGVs (background area).

### 2.9. Biophoton spectral imaging

**Figure 1:**
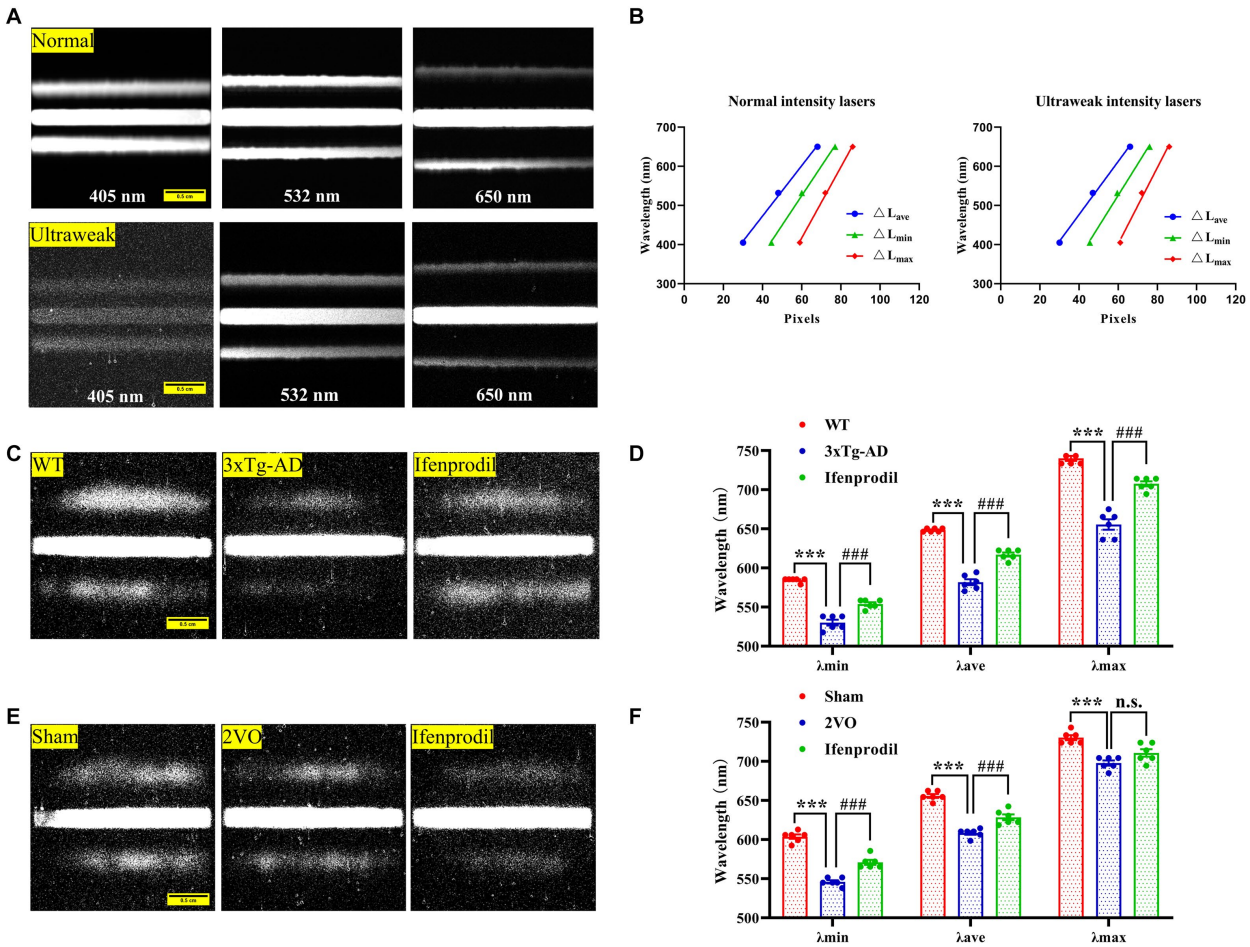
Spectral detection of biophotonic activity. **(A)** Photon spectral images are obtained from three known-wavelength lasers (405 nm, 532 nm, 650 nm) under conditions of normal (up planes) and ultraweak (down planes) light intensities, showing one zero-order fringe and two first-grade fringes. The two first-grade fringes present a trend away from the zero-order fringe from short to long wavelengths. Scale bar: 0.5 cm. **(B)** There are linear relationships between the wavelengths and fringe distances ΔLc, ΔLmin, ΔLmax of three lasers under conditions of normal (left plane) and ultraweak (right plane) light intensities. **(C)** Representative biophoton spectral images in WT, 3xTg-AD and 3xTg-AD + ifenprodil groups. Scale bar: 0.5 cm. **(D)** The spectral range represented by λmin, λave, and λmax in WT, 3xTg-AD and 3xTg-AD + ifenprodil groups. *n* = 6 animals per group. **(E)** Representative biophoton spectral images in sham, 2VO and 2VO + ifenprodil groups. Scale bar: 0.5 cm. **(F)** The spectral range represented by λmin, λave, and λmax in sham, 2VO and 2VO + ifenprodil groups. *n* = 6 animals per group. The data in **(D,F)** were presented as means ± SEMs and analyzed by one-way ANOVA. ***p < 0.001; ^###^*p* < 0.001.

The biophoton spectral detection system (BSDS) mainly consists of three parts, i.e., an UBIS, a biophoton spectral analysis device (BSAD) consisting of transmission grating and a slit, and a biophoton spectral calibration device (BSCD) consisting of a light source relay device, an optical fiber and a light source reduction device. The specific experimental methods were as follows:

1) Calibration of spectral imaging: We obtained photon spectral images from three lasers with known wavelengths (405 nm, 532 nm, 650 nm) in the presence of photons with normal and ultraweak intensities to calibrate spectral imaging (Figure 1A). The distance between the zero-order fringe center and the first-order fringe of the spectral images was measured to calculate the minimum (△Lmin), maximum (△Lmax) and average distance (△Lave). We analyzed the relationships between the average (λave), minimum (λmin), and maximum (λmax) wavelengths and the relative average (△Lave), minimum (△Lmin), and maximum (△Lmax) distances (Supplementary Table S3). The linear regression equations were described as follows:

**Table:** 

λmin(n) = 6.4373ΔLmin + 215.72	(1)
λave(n) = 7.5289ΔLc + 73.504	(2)
λmax(n) = 9.0658ΔLmax − 126.76	(3)
λmin(u) = 6.7939ΔLmin + 205.16	(4)
λave(u) = 8.0068ΔLc + 45.923	(5)
λmax(u) = 9.7389ΔLmax − 181.94	(6)

where *n* represents the normal photon intensities and *u* represents the ultraweak photon intensities.

2) Validation of spectral imaging: We compared four LED light sources (blue: 410–492 nm, green: 450–586 nm, yellow: 555–625 nm, and red: 595–669 nm) detected by the BSDS and the spectrometer (WDS-8, China) to validate spectral imaging. The spectral ranges measured by the two methods were consistent (Supplementary Figure S2; Supplementary Table S4).
3) Biophotonic spectral imaging: The BSAD was adjusted to the focal plane of the imaging lens between the EMCCD and the perfusion chamber. Real-time spectral images were obtained by automatically acquiring an image every 30 min for 540 min, during perfusion with 50 mM glutamate or 10 μM ifenprodil.
4) Image processing and analysis: All original gray images in TIF format were first processed with a program on the MATLAB platform. The biophotonic spectra were analyzed based on the linear regression equations presented above and shown in Table 1. The results are presented as the λave, λmin, and λmax wavelengths.

**Table 1:** Biophotonic spectra in AD and VaD model animals.

Model animals	Groups	Spectra		
		λ_ave_, nm	λ_min_, nm	λ_max_, nm
AD	WT (*n* = 6)	648.43 ± 0.90^***^	584.49 ± 1.13^***^	740.01 ± 3.25^***^
	3xTg-AD (*n* = 6)	581.71 ± 3.79	530.13 ± 3.69	655.61 ± 6.65
	Ifenprodil (*n* = 6)	617.07 ± 2.67^###^	553.91 ± 2.26^###^	707.55 ± 3.25^###^
VaD	Sham (*n* = 6)	655.77 ± 2.46^***^	603.74 ± 2.86^***^	730.27 ± 3.25^***^
	2VO (*n* = 6)	608.40 ± 2.25	545.99 ± 2.09	697.81 ± 3.25
	Ifenprodil (*n* = 6)	628.42 ± 3.69^###^	570.90 ± 3.24^###^	710.79 ± 4.82^n.s.^

WT, wild type group; 3xTg-AD, triple-transgenic Alzheimer’s disease group; 2VO, two-vessel occlusion group.

λave, λmin, and λmax are the average, minimum, and maximum wavelength of biophotonic spectra, respectively. The data were presented as the means ± SEMs and analyzed by one-way ANOVA. ***p < 0.001; ^###^*p* < 0.001; n.s., no significance, *p* > 0.05.

### 2.10. Statistical analyzes

All data were analyzed by GraphPad Prism 8.0 software (GraphPad Software Inc., United States) and presented as the means ± SEMs. Student’s *t* test was applied to analyze differences between two groups, and one-way ANOVA followed by Tukey’s *post hoc* test was used for multiple comparisons. Statistical differences were considered significant when *p* < 0.05 and very significant when *p* < 0.01.

## 3. Results

### 3.1. Cognitive impairments in AD and VaD animals

**Figure 2:**
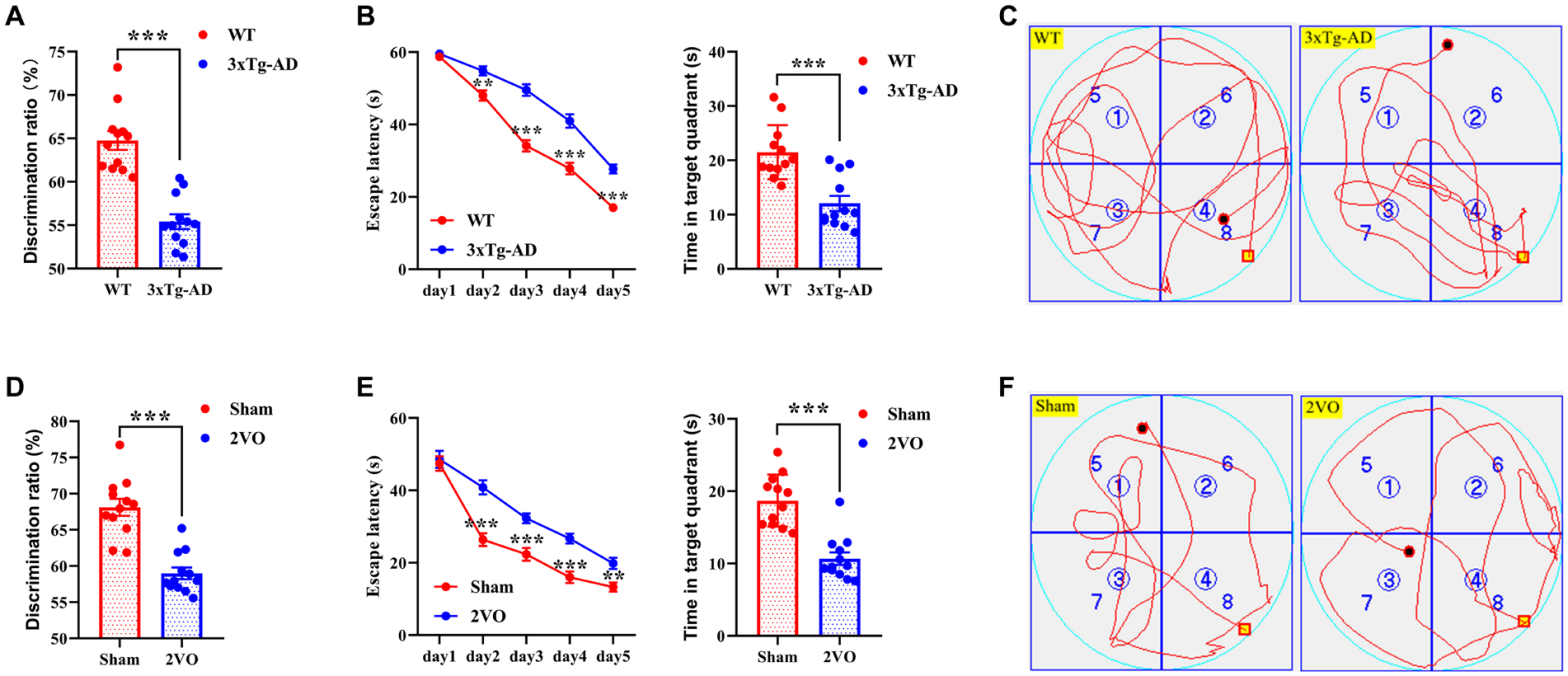
Cognitive impairments in AD and VaD model animals. **(A)** The discrimination ratio of NOR test in WT and 3xTg-AD groups. *n* = 12. **(B)** The escape latency (left plane) and time in target quadrant (right plane) of MWM test in WT and 3xTg-AD groups. *n* = 12. **(C)** Representative swimming track of WT and 3xTg-AD groups during the probe trial. **(D)** The discrimination ratio of NOR test in sham and 2VO groups. *n* = 12. **(E)** The escape latency (left plane) and time in target quadrant (right plane) of MWM test in sham and 2VO groups. *n* = 12. **(F)** The representative swimming track of sham and 2VO groups during the probe trial. The platform in **(C,F)** were located in the northwest quadrant. The data in **(A,B,D,E)** were presented as means ± SEMs and analyzed by Student’s *t* test. **p < 0.01; ***p < 0.001.

The novel object recognition (NOR) test and Morris water maze (MWM) test were performed to assess cognitive functions in AD and VaD model animals. The discrimination ratio of the triple-transgenic Alzheimer’s disease (3xTg-AD) group was significantly lower than that of the wild type (WT) group (Figure 2A), and the discrimination ratio of the two-vessel occlusion (2VO) group was significantly lower than that of the sham group (Figure 2D). Moreover, there was a significant difference in escape latency during the training period and time spent in the target quadrant during the probe trial period between the 3xTg-AD and WT groups and between the 2VO and sham groups, respectively (Figures 2B,E). The representative swimming tracks of animals from each group during the probe trial are shown in Figures 2C,F. Collectively, these data suggest that AD and VaD model animals showed cognitive impairments.

### 3.2. Reduced biophoton emission from synaptosomes in AD and VaD animals

**Figure 3:**
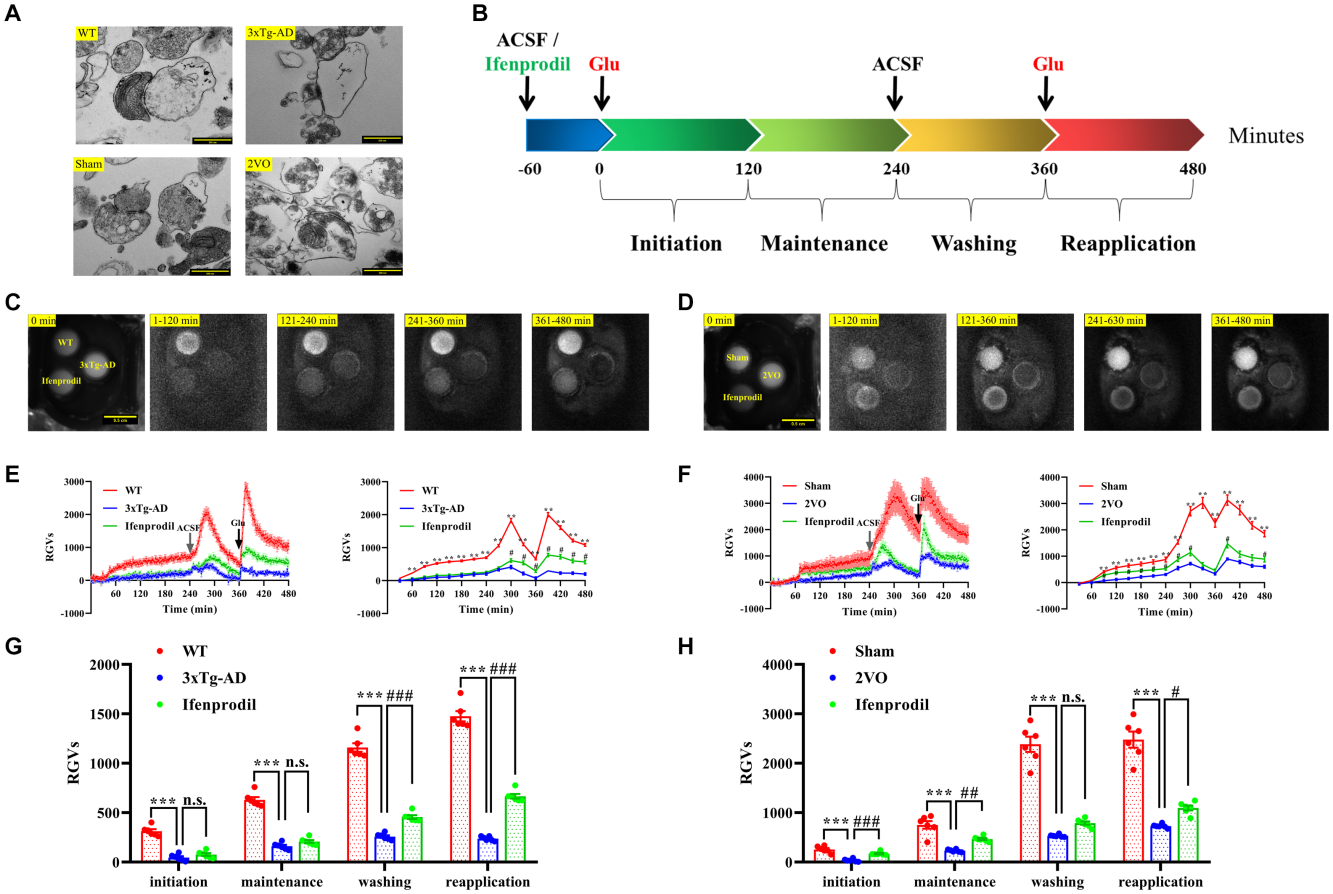
Changes in biophoton emission of synaptosomes after perfusion with glutamate or glutamate + ifenprodil. **(A)** Representative electron microscopy images of the synaptosomes prepared from WT, 3xTg-AD, sham and 2VO groups. Scale bar: 500 nm. **(B)** Experimental scheme for the study of biophton emission induced by 50 mM glutamate or pre-perfusion with 10 μM ifenprodil. **(C)** Representative real-time images of biophoton emission of hippocampal synaptosomes in WT, 3xTg-AD and 3xTg-AD + ifenprodil groups. Each row of representative images from left to right corresponded to positioning, initiation, maintenance, washing, and reapplication periods. Scale bar: 0.5 cm. **(D)** Representative real-time images of biophoton emission of hippocampal synaptosomes in sham, 2VO and 2VO + ifenprodil groups. Scale bar: 0.5 cm. **(E)** The dynamic changes of biophoton emission (left plane) and average values every 30 min (right plane) from synaptosomes in WT, 3xTg-AD and 3xTg-AD + ifenprodil groups were represented by relative gray values (RGVs). *n* = 6 animals per group. **(F)** The dynamic changes of biophoton emission (left plane) and average values every 30 min (right plane) from synaptosomes in sham, 2VO and 2VO + ifenprodil groups were represented by RGVs. *n* = 6 animals per group. **(G)** RGVs of hippocampal synaptosomes in WT, 3xTg-AD and 3xTg-AD + ifenprodil groups during initiation, maintenance, washing, and reapplication periods, respectively. *n* = 6 animals per group. **(H)** RGVs of hippocampal synaptosomes in sham, 2VO and 2VO + ifenprodil groups during initiation, maintenance, washing, and reapplication periods, respectively. *n* = 6 animals per group. The data in **(E–H)** were presented as means ± SEMs and analyzed by one-way ANOVA. **p < 0.01; ***p < 0.001; ^#^*p* < 0.05; ^##^*p* < 0.01; ^###^*p* < 0.001; no significance, *p* > 0.05.

To prepare hippocampal synaptosomes with high purity and complete structure, we used a rapid Percoll gradient method described by Dunkley and colleagues with minor modifications (Dunkley et al., 2008). The synaptosomes isolated from each group had typical morphological and structural characteristics. One or more mitochondria and a large number of synaptic vesicles could be seen in the presynaptic components, and the synaptic clefts and postsynaptic densities were also clearly visible (Figure 3A).

After pre-perfusion with artificial cerebrospinal fluid (ACSF) or 10 μM ifenprodil for 60 min, biophoton emission from resuspended synaptosomes induced with 50 mM glutamate was imaged for 240 min, followed by wash out at 240 min and reapplication of glutamate at 360 min (Figure 3B). The results showed that the biophotonic activities of synaptosomes in each group presented four characteristic periods, which were described as initiation (1–120 min), maintenance (121–240 min), washing (241–360 min), and reapplication (361–480 min) (Figures 3C, D). We found that the biophoton emission from synaptosomes in the 3xTg-AD group was significantly reduced in four periods compared with that in the WT group. Moreover, pre-perfusion of the ifenprodil, a selective antagonist of GluN2B receptors, significantly increased the biophoton emission in washing and reapplication periods of 3xTg-AD group (3xTg-AD + ifenprodil) (Figures 3E,G; Supplementary Table S1). Similarly, the biophoton emission from synaptosomes was significantly reduced in the 2VO group compared with the sham group, and was significantly increased in the initiation, maintenance and reapplication periods of 2VO + ifenprodil group (Figures 3F,H; Supplementary Table S1). These results indicated that the biophoton imaging technology could evaluate synaptic functional changes in the brain of AD and VaD animals.

### 3.3. Reduced biophoton transmission of brain slices in AD and VaD animals

**Figure 4:**
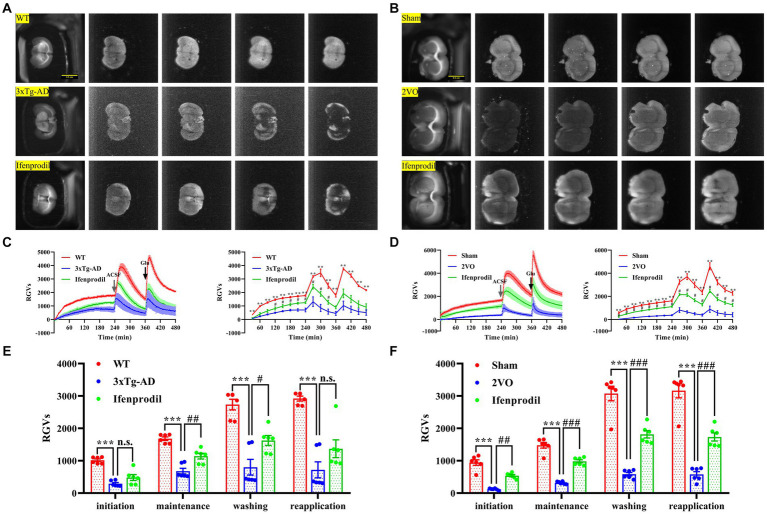
Changes of biophoton transmission in brain slices. **(A)** Representative real-time images of biophoton transmission of brain slices in WT, 3xTg-AD and 3xTg-AD + ifenprodil groups. Each row of representative images from left to right corresponded to positioning, initiation, maintenance, washing, and reapplication periods. Scale bar: 0.5 cm. **(B)** Representative real-time images of biophoton transmission of brain slices in sham, 2VO and 2VO + ifenprodil groups. Scale bar: 0.5 cm. **(C)** The dynamic changes of biophoton emission (left plane) and average values every 30 min (right plane) from brain slices in WT, 3xTg-AD and 3xTg-AD + ifenprodil groups were represented by RGVs. *n* = 6 animals per group. **(D)** The dynamic changes of biophoton emission (left plane) and average values every 30 min (right plane) from brain slices in sham, 2VO and 2VO + ifenprodil groups were represented by RGVs. *n* = 6 animals per group. **(E)** RGVs of brain slices in WT, 3xTg-AD and 3xTg-AD + ifenprodil groups during initiation, maintenance, washing, and reapplication periods, respectively. *n* = 6 animals per group. **(F)** RGVs of brain slices in sham, 2VO and 2VO + ifenprodil groups during initiation, maintenance, washing, and reapplication periods, respectively. *n* = 6 animals per group. The data in **(C–F)** were presented as means ± SEMs and analyzed by one-way ANOVA. ^**^p < 0.01; ^***^p < 0.001; ^#^*p* < 0.05; ^##^*p* < 0.01; ^###^*p* < 0.001; no significance, *p* > 0.05.

The reduced biophoton emission from synaptosomes might be related to changes in the biophotonic activities of neural circuits. Thus, we further detected the biophoton transmission of brain slices in AD and VaD model animals. Four characteristic periods of biophotonic activity were observed in each group (Figures 4A,B). We found that the biophoton transmission of brain slices in the 3xTg-AD group was significantly reduced compared with that in the WT group, and pre-perfusion of ifenprodil increased biophoton transmission of brain slices in the 3xTg-AD + ifenprodil group (Figures 4C,E; Supplementary Table S2). The same results were also found in the sham, 2VO and 2VO + ifenprodil groups (Figures 4D,F; Supplementary Table S2). Remarkably, biophotonic activities of brain slices treated with the pre-perfusion of ifenprodil was significantly increased in the maintenance and washing periods of 3xTg-AD + ifenprodil group but was significantly increased in all four periods of 2VO + ifenprodil group, indicating that the treatment with the pre-perfusion of ifenprodil might partially reverse the reduced biophoton transmission in AD and VaD animals.

### 3.4. Spectral blueshift of biophotonic activities in AD and VaD animals

The biophoton spectral detection system (BSDS) was calibrated by three lasers in the presence of normal and ultraweak light intensities (Figures 1A,B; Supplementary Table S3), and the calculated linear regression equations are described in the methods. Subsequently, the calibrated BSDS was employed to obtain the biophotonic spectra and the data are presented in Table 1. Although the spectral ranges of biophotonic activities in AD and VaD model animals were different (the spectral range in the 3xTg-AD group was 530 ~ 655 nm, while that in the 2VO group was 546 ~ 698 nm), we indeed observed the spectral blueshift of λmin, λave, and λmax in the 3xTg-AD and 2VO groups compared with the WT and sham groups, respectively (Figures 1C–F; Table 1). Interestingly, these changes could also be partially reversed by the pre-perfusion of ifenprodil.

## 4. Discussion

Our previous studies have demonstrated that biophotonic activities contribute to the neural signal transmission and play an important role in brain functions (Tang and Dai, 2014b; Esmaeilpour et al., 2020). Recent research findings have not only verified the feasibility of optical communication in neural circuits but also provided an important basis for establishing a theoretical model of optical communication between nerve fibers (Kumar et al., 2016; Zangari et al., 2018; Zarkeshian et al., 2018; Ambrosetti et al., 2022). Moreover, experimental results have shown that near infrared laser photons induce glutamate release from nerve terminals, and the quantum energy level of glutamate modulate neural biophotonic activity and transmission (Amaroli et al., 2018; Xiang et al., 2020; Han et al., 2021; Maghoul et al., 2021). In the present study, reduced biophotonic activities and spectral blueshift induced by glutamate were found in synaptosomes and brain slices from AD and VaD model animals, indicating that this biophoton imaging technology is accurate and effective in assessing functional changes in synapses and neural circuits. Recently, Sefati et al. detected the ultraweak photon emission (UPE) of rat hippocampus after injection of streptozotocin (STZ) and donepezil via a photomultiplier tubes (PMT) device, suggesting that UPE can be used for screening and diagnosing AD (Sefati et al., 2023). This further supports that the biophoton imaging technology could serve as a non-invasive method for studying brain functions and diseases related to cognitive impairment.

The biophotonic activities may be conducive to transmit and process quantum information in the brain. Myelinated axons are proposed as potential biophoton waveguides in the brain, and ion channel currents in the node of Ranvier behave like an array of nanoantennas emitting biophoton (Kumar et al., 2016; Zangari et al., 2018). Moreover, quantum effects in biological systems have been studied in avian magnetoreception and found that interactions between electron and nuclear spins in cryptochromes could explain how birds perceive the distribution of geomagnetic fields through visual perception (Ritz et al., 2000; Hiscock et al., 2016). The synapses in the neural circuits may provide sufficient contact for coherent interactions between nuclear spins connected to different axons. Therefore, changes in synaptic function in AD and VaD brains probably impair the transmission and processing of biophoton signals.

Synaptic dysfunction is a common pathological feature in complex neurological diseases (Galvin et al., 1999; Milnerwood and Raymond, 2010; Schulz-Schaeffer, 2010; Lu et al., 2013). In this study, we found that the direct biological manifestation of synaptic dysfunction in AD and VD model animals is the reduced biophotonic activities induced by glutamate. One possible explanation for this finding is that the function of NMDA receptors on glutamatergic synapses are altered as previous studies have proven that biophoton emission induced by glutamate is involved in the activity of NMDA receptors (Chai et al., 2018). Additionally, the pre-perfusion of ifenprodil could reverse the decreased biophotonic activity induced by glutamate, implying that the excessive activation of GluN2B may play a role in the functional decline of glutamatergic synaptosomes, and that such a change may occur in the early stage of AD and VaD and is related to the decline in cognitive function, which has been verified by cognitive assessments performed in this study. However, disease progression may involve changes in the number and structure of glutamate receptors and GluN2B subunits, because the expression of GluN2B and GluN2A subunits are selectively changed, especially related to the conversion of GluN2B subunit to GluN2A subunit (Sheng et al., 1994; Okabe et al., 1998; Bi and Sze, 2002; Hynd et al., 2004; Rodenas-Ruano et al., 2012; Bagasrawala et al., 2017), which may result in an overall decline in synaptic function and severe cognitive impairment in the later stage of AD and VaD (Cao et al., 2007).

Functional changes in glutamate receptors, especially GluN2B-containing NMDARs, could also provide an explanation for the spectral blueshift. We speculate that the excessive activation of GluN2B subunits plays a certain role in the emission of the blue spectrum biophoton component in response to glutamate, because the spectral blueshift was found in synaptosomes and brain slices from AD and VaD animals, while the pre-perfusion of ifenprodil could reverse this change. A reasonable explanation based on biophysics is that the blueshifted biophotons consume more energy and are less efficient in transmitting optical information, which may contribute to a decline of cognitive functions in AD and VaD brains. However, the exact biological mechanism remains to be studied.

The hypothesis proposed based on early research suggested that biophotons mainly originate from the generation of various reactive oxygen species (ROS) (Kataoka et al., 2001), but our previous results showed that the cytochrome c oxidase inhibitor (sodium azide) did not completely block the biophotonic activities induced by glutamate (Tang and Dai, 2014b). Moreover, different quantum levels of glutamate had different effects on biophotonic activities (Han et al., 2021), indicating that the transfer of quantum energy during the process of interactions between active molecules such as neurotransmitters, which serve as biological information mediators, plays an important role in the origin of biophotons. The findings of this study provide direct evidence to confirm that such a quantum biological process not only occurs in synapses, but also undergoes significant changes during the pathological process of AD and VaD animal models, providing new ideas for the development of quantum drug therapies for these diseases. Furthermore, we speculate that the background biophotons emitted by reactive oxygen species may contribute to the maintenance of quantum energy levels of active molecules, but this speculation needs to be experimentally verified.

These findings propose a framework for additional studies including: (a) the mechanisms of the reduced biophotonic activities and spectral blueshift; (b) the improvement effect of ifenprodil or similar compounds on cognitive function in early AD and VaD model animals; (c) screening of new drugs for AD and VaD pathology directly targeting on the synaptosomes with biophoton imaging technology.

## 5. Conclusion

This direct study of synaptic functional associated with biophotonic activities provides new methods and evidence for further exploring the pathophysiological mechanism of AD and VaD and new ideas for the development of therapeutic drugs.

## Data availability statement

The original contributions presented in the study are included in the article/Supplementary material, further inquiries can be directed to the corresponding authors.

## Ethics statement

The animal study was approved by Animal Ethics Committee of the Medical School of Wuhan University (No. ZN2021020). The study was conducted in accordance with the local legislation and institutional requirements.

## Author contributions

JjZ, JD, ZW, ZX, and YiL designed the research. ZW, ZX, YiL, SP, HS, TL, JiZ, NL, SW, JuZ, LZ, YH, YpL, and DL performed the research. ZW, ZX, and YiL analyzed the data. JjZ, JD, ZW, and ZX wrote and revised the paper. All authors contributed to the article and approved the submitted version.

## Funding

This research was supported by National Natural Science Foundation of China (nos. 82071210, 31700911, 82071324, and 82271232), the innovation team fund of National Ethnic Affairs Commission (MZR20002) and South-Central Minzu University (KTZ20039 and CZP18003).

## Conflict of interest

The authors declare that the research was conducted in the absence of any commercial or financial relationships that could be construed as a potential conflict of interest.

## Publisher’s note

All claims expressed in this article are solely those of the authors and do not necessarily represent those of their affiliated organizations, or those of the publisher, the editors and the reviewers. Any product that may be evaluated in this article, or claim that may be made by its manufacturer, is not guaranteed or endorsed by the publisher.
