# Supplementary material for: Reduced biophotonic activities and spectral blueshift in Alzheimer’s disease and vascular dementia models with cognitive impairment
Source: Front Aging Neurosci. 2023 Sep 1;15:1208274. doi: 10.3389/fnagi.2023.1208274 (PMC10505668; doi:10.3389/fnagi.2023.1208274)
Supplement: Supplementary file 1 [file Data_Sheet_1.DOCX]

Supplementary Material

Reduced biophotonic activities and spectral blueshift in Alzheimer's disease and vascular dementia models with cognitive impairment

Zhuo Wang^1,2†^, Zhipeng Xu^1†^, Yi Luo^3^, Sisi Peng^1^, Hao Song^1^, Tian Li^1^, Jiaxin Zheng^1^, Na Liu^2^, Shenjia Wu^1^, Junxia Zhang^4^, Lei Zhang^1^, Yuan Hu^1^, Yanping Liu^1^, Dongwei Lu^1^, Jiapei Dai^2*^ and Junjian Zhang^1*^

*** Correspondence:**

Junjian Zhang
[wdsjkx@163.com](mailto:wdsjkx@163.com)

Jiapei Dai

[jdai@mail.scuec.edu.cn](mailto:jdai@mail.scuec.edu.cn)

# Supplementary Figures

**
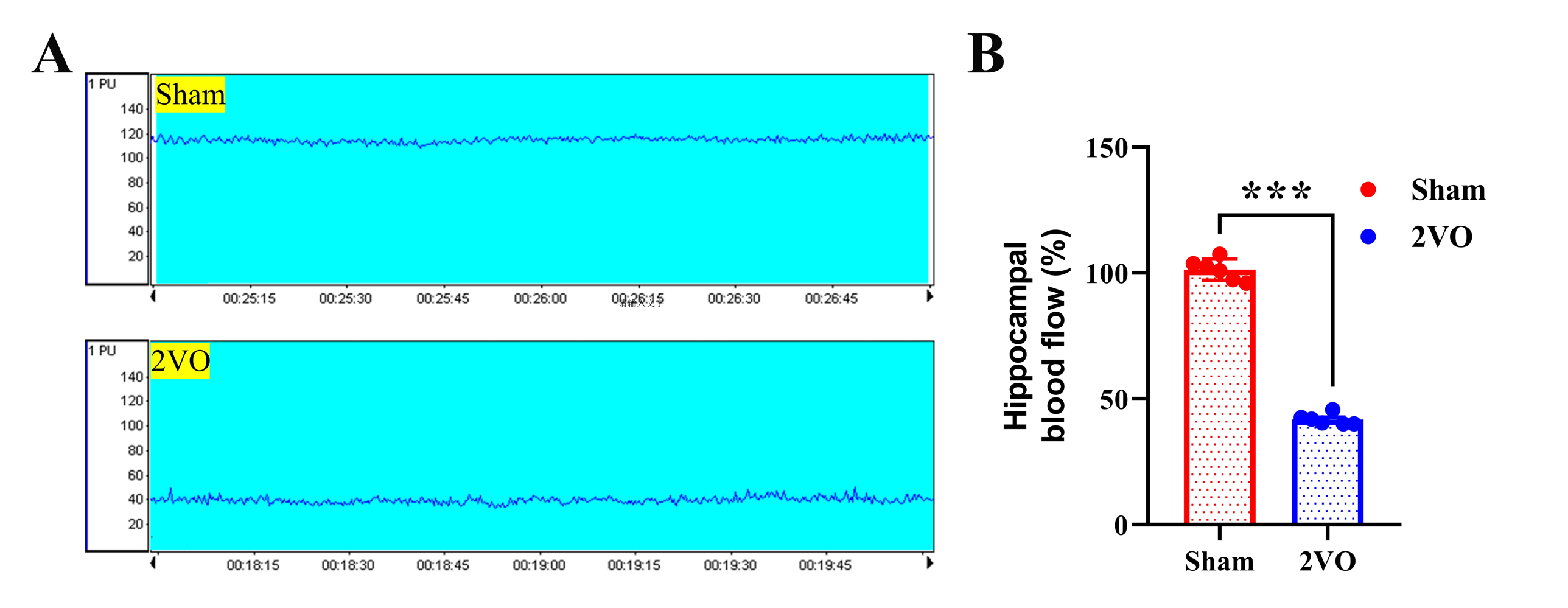
**

**Supplementary FIGURE S1**

Hippocampal blood flow in CCH model animals. **(A)** Representative hippocampal blood flow in CA1 area in sham and 2VO operation groups. **(B)** Changes of hippocampal blood flow in sham and 2VO groups. *n* = 6 animals per group.

The data in **B** were presented as means ± SEMs and analyzed by Student’s t test. ****p* < 0.001.

**
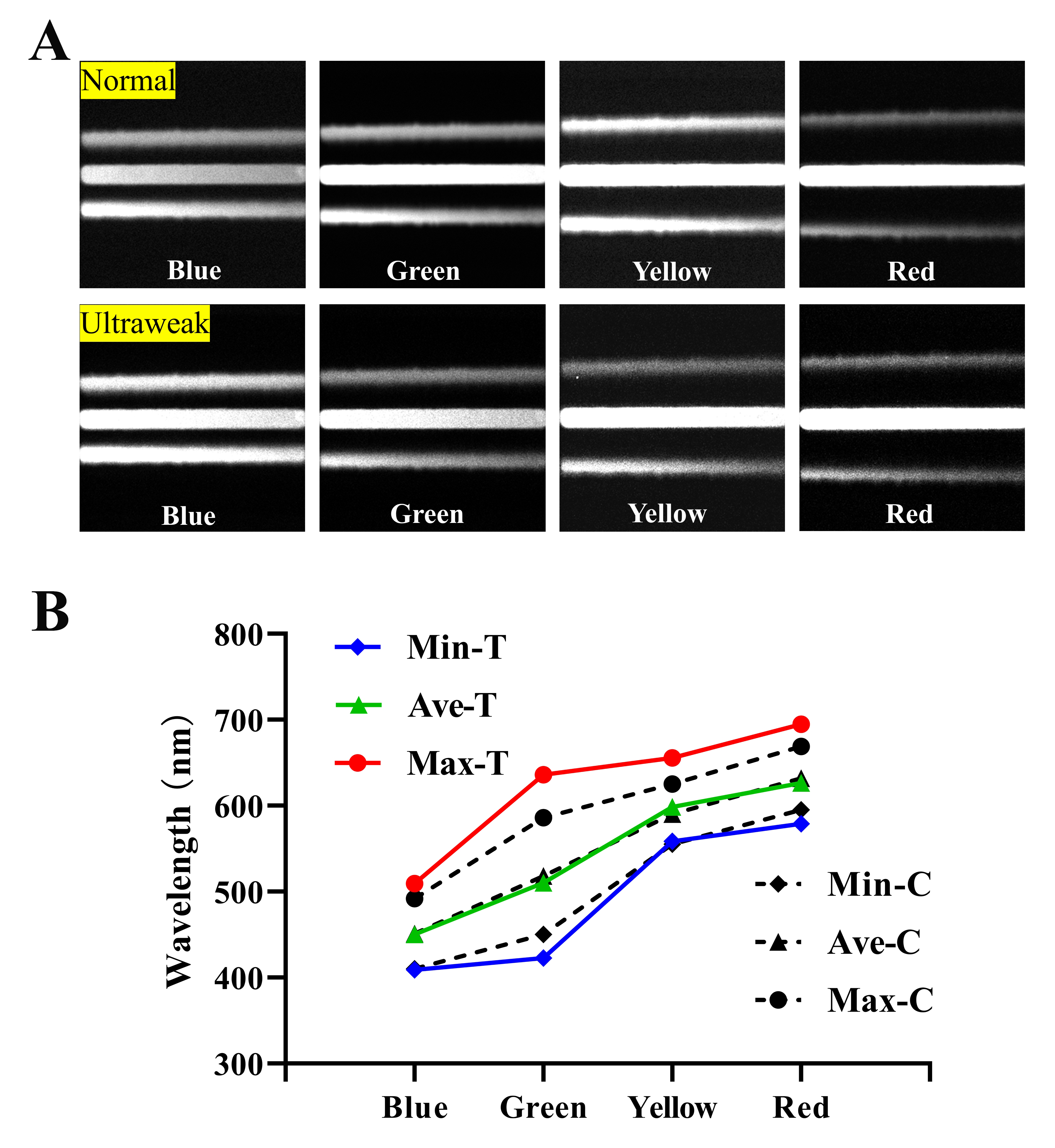
**

**Supplementary FIGURE S2**

Validation of the BSDS by four LED light sources. (A) Photon spectral images were obtained from four LED lights (blue, green, yellow, and red) using the biophoton spectral detect system (BSDS) under conditions of normal (up planes) and ultraweak (down planes) light intensities. The first-grade fringe presented a trend away from the zero-order fringe from blue to red LED light. (B) The relationship between the wavelengths of the LED light (λave, λmin and λmax) calculated from the spectral images (color symbols) and the spectral range detected by the spectrometer (black symbols). The calculated λave is almost the same as the known peak wavelength of each LED light.

# Supplementary Tables

**Supplementary Table S1.** RGVs of biophoton emission from synaptosomes. Related to **FIGURE 2.**

.

| Model animals | Groups | RGVs | | | |
| --- | --- | --- | --- | --- | --- |
|  |  | **initiation** | **maintenance** | **washing** | **reapplication** |
| AD | WT (*n* = 6) | 312.58 ± 20.57 **^***^** | 629.79 ± 28.39 **^***^** | 1159.46 ± 43.06 **^***^** | 1475.81 ± 51.63 **^***^** |
|  | 3xTg-AD (*n* = 6) | 45.99 ± 13.52 | 161.30 ± 13.62 | 257.92 ± 13.12 | 237.29 ± 9.55 |
|  | Ifenprodil (*n* = 6) | 76.03 ± 13.98 ^n. s.^ | 208.01 ± 14.46 ^n. s.^ | 455.27 ± 18.73 ^###^ | 665.87 ± 23.42 ^###^ |
| VaD | Sham (*n* = 6) | 256.09 ± 27.06 **^***^** | 751.29 ± 77.81 **^***^** | 2383.50 ± 155.24 **^***^** | 2478.24 ± 162.38 **^***^** |
|  | 2VO (*n* = 6) | 34.64 ± 11.89 | 230.20 ± 11.81 | 531.74 ± 10.36 | 728.41 ± 15.99 |
|  | Ifenprodil (*n* = 6) | 169.21 ± 14.57 ^###^ | 466.55 ± 21.98 ^##^ | 788.28 ± 34.16 ^n. s.^ | 1095.45 ± 50.12 ^#^ |

Abbreviations: WT, wild type group; 3xTg-AD, triple-transgenic Alzheimer’s disease group; 2VO, two-vessel occlusion group.

The data were presented as the means ± SEMs and analyzed by one-way ANOVA. ****p* < 0.001; ^#^*p* < 0.05; ^##^*p* < 0.01; ^###^*p* < 0.001; n.s., no significance, *p* > 0.05.

**Supplementary Table S2.** RGVs of biophoton transmission in brain slices. Related to **FIGURE 3.**

.

| Model animals | Groups | RGVs | | | |
| --- | --- | --- | --- | --- | --- |
|  |  | **initiation** | **maintenance** | **washing** | **reapplication** |
| AD | WT (*n* = 6) | 1013.73 ± 40.52 **^***^** | 1680.69 ± 54.98 **^***^** | 2734.92 ± 164.84 **^***^** | 2920.89 ± 74.38 **^***^** |
|  | 3xTg-AD (*n* = 6) | 290.483 ± 5.58 | 676.88 ± 86.68 | 799.89 ± 241.01 | 719.77 ± 246.49 |
|  | Ifenprodil (*n* = 6) | 482.709 ± 0.46 ^n.s.^ | 1141.64 ± 88.09 ^##^ | 1625.23 ± 152.59 ^#^ | 1369.11 ± 275.36 ^n.s.^ |
| VaD | Sham (*n* = 6) | 943.43 ± 86.17 **^***^** | 1479.68 ± 86.68 **^***^** | 3075.81 ± 224.21 **^***^** | 3164.57 ± 221.38 **^***^** |
|  | 2VO (*n* = 6) | 122.19 ± 10.27 | 319.88 ± 16.81 | 584.45 ± 52.06 | 577.06 ± 85.19 |
|  | Ifenprodil (*n* = 6) | 537.82 ± 32.94 ^##^ | 982.60 ± 46.55 ^###^ | 1814.98 ± 114.66 ^###^ | 1733.29 ± 124.58 ^###^ |

The data were presented as the means ± SEMs and analyzed by one-way ANOVA. ****p* < 0.001; ^#^*p* < 0.05; ^##^*p* < 0.01; ^###^ *P* < 0.001; n.s., no significance, *p* > 0.05.

**Supplementary Table S3.** Linear correlation between laser spectra and ΔLmin, ΔLc，ΔLmax. Related to **FIGURE 4**.

| Light intensity | Spectra | ΔLmin, pixels | ΔLc, pixels | ΔLmax, pixels |
| --- | --- | --- | --- | --- |
| Normal | 405 nm | 30 | 44.5 | 59 |
|  | 532 nm | 48 | 60 | 72 |
|  | 650 nm | 68 | 77 | 86 |
| Correlation analysis | *R*² | 0.9973 | 0.9977 | 0.9982 |
|  | *P* | 0.0328 | 0.0305 | 0.0271 |
| Ultraweak | 405nm | 30 | 45.5 | 61 |
|  | 532nm | 47 | 59.5 | 72 |
|  | 650nm | 66 | 76 | 86 |
| Correlation analysis | *R*² | 0.9972 | 0.9953 | 0.9919 |
|  | *P* | 0.0339 | 0.0436 | 0.0475 |

Each value of ΔLmin, ΔLc and ΔLmax represents the average of the three repeated tests.

**Supplementary Table S4.** Spectra of four LED light source detected by BSDS. Related to **Supplementary FIGURE S2**.

| LED light source | Peak, nm | λ_ave_, nm | start, nm | λ_min_, nm | stop, nm | λ_max_, nm |
| --- | --- | --- | --- | --- | --- | --- |
| Blue | 451 | 450.3 | 410 | 409.0 | 492 | 509.5 |
| Green | 518 | 510.3 | 450 | 422.6 | 586 | 636.1 |
| Yelow | 590 | 598.4 | 555 | 558.4 | 625 | 655.6 |
| Red | 632 | 626.4 | 595 | 578.8 | 669 | 694.6 |

Start, peak and stop represent the spectral range of four LED lights detected by the spectrometer, respectively. λave, λmin and λmax represent the wavelengths calculated from the spectral images under the conditions of ultraweak light intensities.
